# The Antifungal Activity of a Polygalacturonic and Caprylic Acid Ointment in an In Vitro, Three-Dimensional Wound Biofilm Model

**DOI:** 10.3390/jof11030178

**Published:** 2025-02-24

**Authors:** Bahgat Z. Gerges, Joel Rosenblatt, Y-Lan Truong, Ying Jiang, Issam I. Raad

**Affiliations:** Department of Infectious Diseases, Infection Control and Employee Health Research, The University of Texas MD Anderson Cancer Center, 1515 Holcombe Blvd, Houston, TX 77030, USA; jsrosenblatt@mdanderson.org (J.R.); yltruong@mdanderson.org (Y.-L.T.); yijiang@mdanderson.org (Y.J.); iraad@mdanderson.org (I.I.R.)

**Keywords:** polygalacturonic acid, caprylic acid, wound biofilm, *Candida albicans*, *Candida tropicalis*, *Candida parapsilosis*, *Candida glabrata*, *Candida auris*

## Abstract

*Candida* colonization and biofilms are significant contributors to impaired wound healing. Consequently, improved treatments are needed to eradicate *Candida* biofilms in wounds. Wounds present complex biofilm extracellular matrix environments, with microbial cells frequently enmeshed in matrices comprising wound exudate macromolecular gels. We evaluated the ability of a polygalacturonic and caprylic acid (PG + CAP) ointment to eradicate *Candida albicans*, *C. parapsilosis*, *C. glabrata*, *C. tropicalis*, and *C. auris* biofilms in a fibrin gel wound biofilm model of the complex wound biofilm environment. Hypochlorous acid (HOCl) is a disinfecting antimicrobial agent that is widely used as wound irrigant, and this was used as a comparator. A single treatment with PG + CAP reduced the number of viable organisms in the *C. albicans* and *C. glabrata* biofilms by over 5 log_10_, in the *C. parapsilosis* and *C. auris* biofilms by over 4 log_10_, and in the *C. tropicalis* biofilm by 3.85 log_10_. PG + CAP was superior (*p* < 0.01) to HOCl in eradicating all *Candida* species biofilms, except for *C. auris*, for which both treatments fully eradicated all viable organisms. The use of HOCl in *Candida*-colonized wounds should include consideration of the extracellular matrix load in the wound bed. PG + CAP warrants further study in wounds compromised by *Candida* biofilms.

## 1. Introduction

Delayed- or non-healing wounds are a major human health problem. It has been estimated that over 10 million Americans suffer from chronic wounds, and that chronic wounds impact the lives of 2.5% of the population [1]. Additionally, the financial burden of wounds on the health care system is substantial; an estimated 5% of the entire Medicare budget is spent on wound care, and an estimated 3% of United Kingdom health care spending is consumed by wound care [2,3]. The microbial colonization of wound beds and the formation of recalcitrant biofilms are significant actors in preventing or delaying closure and healing of full-thickness dermal wounds [4]. Due to their ability to colonize human skin, *Candida* species are important pathogens involved in wound biofilms. These fungal biofilms can delay healing by causing a persistent inflammatory response, which leads to high levels of proteases that dissolve newly deposited granulation tissue. In a survey of over 900 chronic wounds, 23% were found to be colonized by *Candida* species, the most prevalent being *C. albicans*, *C. parapsilosis*, *C. tropicalis*, and *C. glabrata* [5]. *Candida auris* is an emerging yeast pathogen of major concern, because of its ability to cause hospital outbreaks of invasive candidiasis and to develop resistance to antifungal drugs. The majority of *C. auris* isolates are resistant to fluconazole, an azole drug used for the treatment of invasive candidiasis [6,7]. *Candida auris*, as well as echinocandin-resistant *C. parapsilosis* and *C. glabrata*, are worrisome pathogens with increasing prevalence all over the world and high attributable morbidity and mortality [8,9]. *Candida auris* can colonize skin and wounds and can be spread environmentally. The incidence of *Candida* infections in wounds including *C. auris* is likely to increase [10,11].

Given the potential of *Candida* species to form biofilms, as well as increasing antifungal resistance, improved antifungal treatments are needed that can rapidly eradicate *Candida* biofilms in wound beds without inducing antifungal resistance or inflammation. Antibiotic and antifungal wound ointments pose risks of developing resistance when biofilms are present. Antiseptic wound ointments are broad-spectrum, and do not encourage the development of antimicrobial-resistant microorganisms, but present toxicities to fibroblasts and other mammalian cells involved in the wound healing process. The toxicities can induce inflammatory responses, which can delay wound healing by degrading newly deposited granulation tissue [12,13,14]. Alternatives to antiseptic antifungal and antibiotic ointments are needed that can eradicate wound biofilms without inducing resistance or inflammatory responses. Natural plant-based agents have been shown to provide optimal biofilm disinfection without leading to antimicrobial resistance or toxicity [15] and are the sources for the PG and CAP ointment studied here. This has led to increasing interest and study of non-antiseptic and non-antifungal plant-based wound therapies. Caprylic acid (CAP), a medium-chain fatty acid, has been reported to have potent antifungal activity against *Candida* species by disrupting cell membrane integrity [16,17,18]. In previous studies, polygalacturonic acid (PG) potentiated the antimicrobial activity of CAP by maintaining an optimal antimicrobial pH and by emulsifying CAP into microdroplets that more effectively penetrated and eradicated microbes in biofilm [19,20]. In addition, PG was shown to mediate other beneficial wound healing activities by preventing dehydration of wound beds and partially inhibiting proteases that are destructive to newly deposited granulation tissue [21]. Hypochlorous acid (HOCl) is a widely used antimicrobial wound irrigant that rapidly kills microbes through oxidative chemical reactions without inducing antifungal resistance and was used as a comparator [22].

Traditional biofilm models provide colonization of surfaces by microbes bathed in nutrient broths. This is a suitable model for medical devices or environmental surfaces; however, wounds provide more complex biofilm environments where microbes colonize three-dimensional matrices of proteins and other extracellular molecules that are present at the surfaces of wound beds. Frequently, the matrices at the surfaces of open wounds comprise condensed components of wound exudate that are rich in fibrin [23]. Therefore, we studied the comparative antifungal efficacies of a novel PG + CAP ointment and the widely used comparator wound irrigant HOCl (400 ppm) against the multiple relevant wound *Candida* species in a three-dimensional fibrin gel wound biofilm model.

## 2. Material and Methods

Antifungal Agents: HOCl (Aqua Science Inc., Columbus, OH, USA. Manufacturer/Supplier: Sani-TEST LLC, Slatington, PA, USA. Catalogue # 01S.06E, CAS # 7790-92-3) wound solution was used directly at a dilution of 400 ppm, and 1% PG + 0.8% CAP ointment was prepared in a laboratory, as previously described by Gerges et al., 2021 [20]. PG (Polygalacturonic acid. Sigma-Aldrich Inc., St. Louis, MO, USA. Made in Switzerland. Catalogue # P3889-100G, CAS # 25990-10-7), CAP (Octanoic acid. Sigma-Aldrich Inc., St. Louis, MO, USA. Catalogue # 03907, CAS # 124-07-2). The ointment base for PG + CAP was an aqueous gel containing 2-hydroxyethylcellulose (Sigma-Aldrich Inc., St. Louis, MO, USA. Catalogue # 434973-250G, CAS # 9004-62-0) and glycerol (Fisher Chemical. Fisher Scientific. Janssen Pharmaceutical, Raritan, NJ, USA. Catalogue # G33-500, CAS No. 56-81-5).

*Candida* Isolates: A biofilm eradication assay was conducted using the US Centers for Disease Control and Prevention and US Food and Drug Administration highly resistant clinical isolates of *C. tropicalis* (MDA #112), *C. glabrata* (ATTC #2950), *C. parapsilosis* (MDA #113), *C. albicans* (MDA #117), and *C. auris* (AR #0391). For testing, the organisms were grown from glycerol stock and streaked on Sabouraud dextrose agar (SDA) (Fisher Scientific. Remel Inc., Lenexa, KS, USA. Catalogue # R01766), and then incubated aerobically at 37 °C for 1–2 days to grow. Each organism was cultured separately. After growth, pure colonies from SDA were picked up by a sterile loop and inoculated into Mueller Hinton broth (MHB) (Fisher Scientific. BBL Mueller Hinton II Broth Cation Adjusted. Sparks, MD, USA. Catalogue # B12322), and then incubated at 37 °C for 1–3 h for fungal refreshment. Each isolate was diluted to 0.5 McFarland using phosphate buffer saline (PBS) (Fisher Scientific. USA. Catalogue # BP3991) Further dilutions were made as necessary for testing.

Three-Dimensional Fibrin Gel Wound Biofilm Model: In the current study, we used a quantitative in vitro three-dimensional wound biofilm model adapted from the model of Besser and Stuermer 2019 [24]. Fibrinogen (20 mg/mL) (Fisher Scientific, Waltham, MA, USA. Catalogue # 34-157-61GM) slowly dissolved in phosphate-buffered saline at 37 °C, thrombin (5 units/mL) (Sigma-Aldrich, St. Louis, MO, USA. Catalogue # T7009-100UN; CAS # 9002-04-4) in phosphate-buffered saline, and calcium chloride (125 mM) (Sigma-Aldrich Inc., Millipore Sigma, Burlington, MA, USA. Catalogue # C3306-100G, CAS # 10035-04-8) in deionized water, and this solution was prepared as described by Truong et al. (2022) [25]. In the current study, we compared the efficacies of PG + CAP and HOCl against 5 different *Candida* species using the FGWB model.

**Figure 1:**
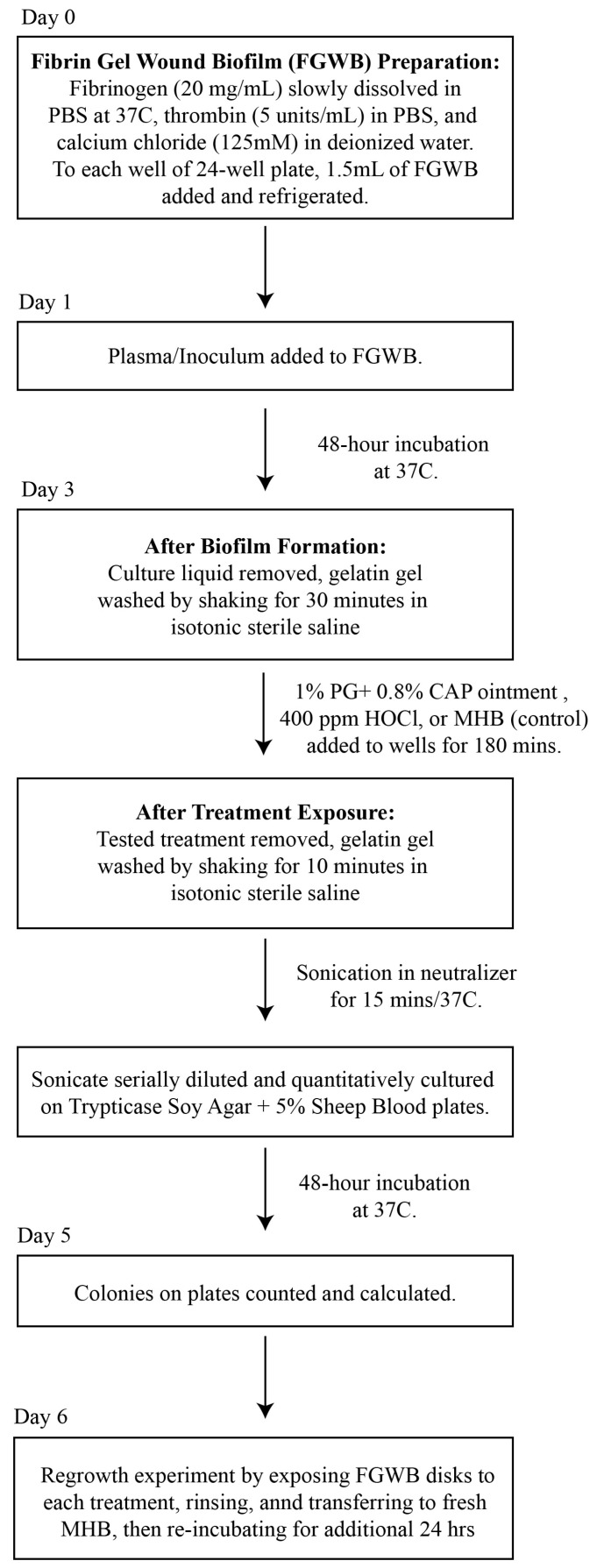
A flow diagram for the preparation of the FGWB model, and the procedures for the biofilm eradication assay. Abbreviations: FGWB, fibrin gel wound biofilm; PBS, phosphate buffer saline; PG + CAP, polygalacturonic acid + caprylic acid; HOCl, hypochlorous acid; MBH, Mueller Hinton broth.

A flow diagram for the preparation of the FGWB model used to evaluate the eradication of *Candida* biofilms following exposure to PG + CAP or HOCl is presented in Figure 1. Briefly, 1.5 mL of fibrin gel (described above) was added to each well of 24-well flat-bottom cell culture plates and exposed to 1.5 mL of human plasma containing 10^4^ colony-forming units of tested *Candida* isolate per milliliter (CFU/mL), and then incubated for 48 h at 37 °C, forming disk-shaped biofilms. Forty-eight hours was selected as the incubation duration to ensure that mature biofilms had formed for each *Candida* species. All culture liquids were then removed, and the colonized fibrin gel disks were washed for 30 min in isotonic sterile saline to remove any remaining planktonic organisms.

Biofilm Eradication Assay: After washing, the resulting FGWB disks were exposed to PG + CAP or HOCl, and a negative control was assessed by adding Mueller Hinton broth, followed by incubation at 37 °C for 180 min. The exposure duration was selected to simulate a reasonable exposure time in a three-dimensional wound bed, before the treatment might be appreciably diluted by wound exudate. After exposure to the treatments, the FGWB disks were washed for 10 min to remove any excess of the tested solutions. The viable organisms remaining on the surface of the FGWB disks were assessed by disrupting the biofilm by sonicating the disks in 5 mL of neutralizer (Fisher Scientific, Waltham, MA, USA. Catalogue # DF0819-17-2) for 15 min. The resulting sonicate was serially diluted with PBS and quantitatively cultured onto SDA. Six replicates each of PG + CAP, HOCl, and negative control were used for each organism. To ensure that eradication was complete (no surviving dormant or persister cells) on FGWB disks from which no viable colonies were recovered following exposure to PG + CAP or HOCl, we conducted regrowth experiments by first exposing the FGWB disks to each experimental solution, then rinsing, and subsequently transferring the disks to fresh MHB and re-incubating for an additional 24 h. Following the 24 h regrowth interval, FGWB disks were sonicated and cultured, as indicated above, to determine whether any organisms remaining embedded in the biofilm were still viable.

Statistical Analyses: The Shapiro-Wilk test was used to check whether data were normally distributed. Then, to compare the antifungal efficacy among the three treatments (PG + CAP, HOCl, and negative control), we performed the non-parametric Kruskal–Wallis test on the CFU/mL values from six replicates for each *Candida* isolate, due to the lack of normality in the data. If a significant difference was found from a three-group comparison, then pairwise comparisons were performed using the Wilcoxon rank sum test. To control the overall type I error for multiple comparisons, the *p*-values for the pairwise comparisons were adjusted using Holm’s sequential Bonferroni procedure. All the tests were two-sided, with a significance level of 0.05. Additionally, the log_10_ reduction in CFU/mL was calculated between the negative control and PG + CAP, and between negative control and HOCl. The statistical analyses were performed using SAS version 9.4 (SAS Institute Inc., Cary, NC, USA).

## 3. Results

**Figure 2:**
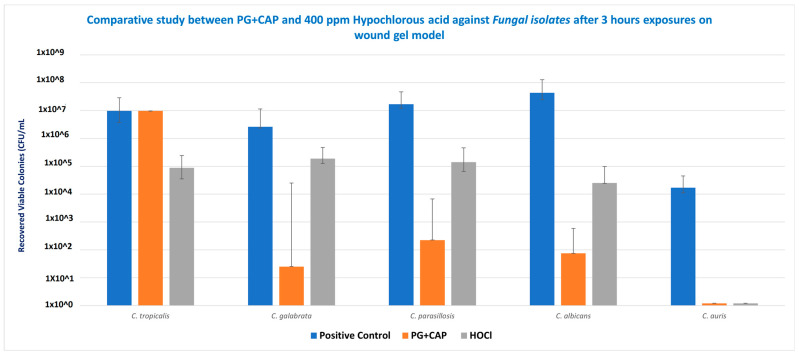
Eradication of biofilms from representative infectious pathogens *Candida tropicalis*, *C. glabrata*, *C. parapsilosis*, *C. albicans*, and *C. auris* by 1% polygalacturonic acid + 0.8% caprylic acid (PG + CAP), compared with 400 ppm hypochlorous acid (HOCl) wound irrigant, after 3 h of exposure. Nontreated fibrin gel biofilm disks were used as negative control. Data are presented as median recovered viable colonies; bars indicate range. CFU, colony-forming units.

Table 1 tabulates the medians for the six replicates for each species for the control group, and the calculated log_10_ reductions for each treatment relative to the control. Figure 2 presents the medians and ranges for the six replicates for each treatment group for *Candida* species in the biofilm eradication experiment in the FGWB model. The *p*-values calculated from the statistical analysis for the log_10_ reductions of PG + CAP versus HOCl for each species are presented as well. After 3 h of exposure, PG + CAP was able to reduce viable *C. tropicalis* by 3.85 log_10_, and HOCl reduced viable *C. tropicalis* by 2.01 log_10_, relative to the control. PG + CAP and HOCl reduced viable *C. glabrata* by 5.02 log_10_ and 1.15 log_10_, respectively, relative to the negative control. PG + CAP and HOCl reduced *C. parapsilosis* by 4.88 log_10_ and 2.09 log_10_, respectively, relative to the control, and reduced *C. albicans* by 5.76 log_10_ and 3.23 log_10_, respectively, relative to control. Both PG + CAP and HOCl were able to completely eradicate *C. auris* after the same period of incubation (4.16 log_10_). The antimicrobial superiority of PG + CAP relative to HOCl was statistically significant (*p* ≤ 0.05) against *C. tropicalis*, *C. glabrata*, *C. parapsilosis*, and *C. albicans*. Although *p*-values are not presented in Table 1, the log_10_ reductions for both PG + CAP versus the control and HOCl versus the control were statistically significant for all *Candida* species tested (*p* ≤ 0.05).

## 4. Discussion

In vitro antifungal efficacy testing in the FGWB model is expected to correspond to the in vivo environment of wound beds more closely than other biofilm models [26]. The FGWB model presents a biofilm in a three-dimensional matrix, as opposed to on a flat surface, and is embedded in a protein matrix rich in a prominent wound exudate protein. To eradicate the *Candida* biofilm in this matrix, the antifungal agents need to penetrate and survive contact with both the fibrin and the *Candida* extracellular biofilm before acting on the embedded *Candida* cells. As shown in Figure 2, following the 3 h treatment duration, both HOCl and PG + CAP partially eradicated the *Candida* cell microbes embedded in the biofilm within the fibrin gels, except for *C. auris*, for which both PG + CAP and HOCl completely eradicated the organisms. HOCl reduced viable fungal cells in *C. tropicalis*, *C. glabrata*, and *C. parapsilosis* biofilms by only 1–2 log_10_ (10–100-fold) compared with the control. *C. albicans* viable cell counts were reduced by over 3 log_10_ (over 1000-fold), and *C. auris* was fully eradicated. PG + CAP reduced viable fungal cells in *C. glabrata*, *C. parapsilosis*, *C. albicans*, and *C. auris* biofilms by over 4 log_10_ (by over 5 log_10_ for *C. glabrata* and *C. albicans*), and by nearly 4 log_10_ (3.85 log_10_) in *C. tropicalis* biofilm. *C. auris* was fully eradicated during the treatment interval. Control *Candida* biofilms had fungal cell counts of well over 10^6^ CFU/mL, except for *C. auris*, for which the cell count was only 1.6 × 10^4^ CFU/mL. As shown in Table 1, PG + CAP was superior (*p* ≤ 0.05) to HOCl against all strains of *Candida* except *C. auris*, against which both were equally effective. Noticeably, all tested strains of *Candida* species had grown well on SDA after 48 h incubation at 37 °C.

The colonization and formation of biofilm by the relevant *Candida* species in the fibrin gels initially required contact and attachment to the fibrin molecules comprising the gel matrix. *Candida albicans* has been reported to have a specific cell surface fibrinogen-binding mannoprotein to facilitate the attachment and colonization process [27]. *Candida albicans* had the highest cell density in the fibrin gel biofilm (greater than 10^7^ colonies/mL), possibly due to its fibrinogen-specific binding adhesin. *Candida glabrata* has also been reported to express specific adhesins for molecules present in wound extracellular matrices [28]. *Candida parapsilosis* and *C. tropicalis* have also been reported to express similar adhesins [29,30]. *Candida tropicalis*, *C. glabrata*, and *C. parapsilosis* presented relatively high cell densities in their fibrin gel biofilms (greater than 10^6^ CFU/mL), but not quite as high as that of the *C. albicans* biofilm. In contrast, *C. auris* had much lower densities in the fibrin gel biofilm (only slightly greater than 10^4^ CFU/mL) than the other *Candida* species. *Candida auris* is reported to have a unique adhesin (SCF1) that mediates surface association and colonization. SCF1 is reported to rely on cationic residues for surface association [31,32]. Fibrinogen and fibrin have an isoelectric pH of 5.5, and, hence, are anionic in the alkaline environments reported to be present in chronic wounds, which might require *C. auris* to bind to matrix molecules other than fibrin in order to attach and aggressively colonize the wound [33,34].

HOCl is a strong chemical oxidizing agent that rapidly reacts with oxidizable substrates [35]. As a very small molecule, HOCl can rapidly penetrate matrices by diffusion, and react nonspecifically with any oxidizable moiety. HOCl molecules are fully consumed during their oxidative reactions and are no longer able to fulfill an antifungal function once oxidized. In a complex extracellular biofilm matrix embedded in a fibrin gel, HOCl contacts many oxidizable moieties before encountering *Candida* cells. Hence, due to the nonspecific oxidative activity of HOCl, its effective concentration can be substantially diminished by the time it reaches *Candida* cells in the FGWB model, and its ability to eradicate these cells is therefore limited. This may, in part, explain the reduced efficacy of HOCl against *C. tropicalis*, *C. glabrata*, *C. parapsilosis*, and *C. albicans*, as shown in Figure 2. This finding is consistent with that of Rembe et al., 2020 [35], whose human plasma biofilm model similarly required HOCl to penetrate matrices replete with oxidizable moieties before contacting microbial cells [35,36]. In contrast, *C. auris* in the FGWB model presented both fewer cells and perhaps a less dense extracellular matrix, thus enabling HOCl to reach *C. auris* cells with sufficient intact concentration to fully eradicate them.

PG + CAP, in contrast to HOCl, exerts its antimicrobial effect by a physical–chemical action, rather than by a chemical reaction. As previously mentioned, CAP exerts its antifungal effect by disrupting cell membranes through a detergent-like effect [17,18]. The extent of membrane damage is enhanced when CAP is combined with an organic acid such as PG, and the microbicidal activity of CAP is further increased when it is delivered to cell surfaces in microdroplets by an emulsifier such as PG [37]. PG + CAP was therefore able to penetrate the extracellular biofilm matrix in the *Candida* biofilms and eradicate fungal cells more potently than HOCl, due to the higher effective PG + CAP concentrations presented at the *Candida* cell surfaces. From the standpoint of tolerability to human cells, flow cytometry studies have shown that CAP is the least cytotoxic fatty acid [38].

In conclusion, we have found that in the wound-mimetic environment of a fibrin gel biofilm, PG + CAP was significantly more effective than HOCl at eradicating *Candida* species. The FGWB model is likely more representative of the wound environment than traditional biofilm models; however, our study remains limited by its use of an in vitro model, which might not have completely captured the entire complexity of a chronic wound biofilm in vivo. Additional study is required to better correlate quantitative reductions in biofilm viability in the model with clinical benefits. This pilot study has additional limitations. Although highly resistant CDC- and FDA-sourced isolates were utilized as worst-case pathogens, testing of additional biological replicates is warranted. We tested each isolate independently, while wound biofilms are complex and frequently involve polymicrobial colonization, which was not assessed here. Although PG + CAP appears promising, it requires more in vivo testing for its efficacy in eradicating *Candida* biofilms in chronic wounds. The use of HOCl to disinfect wounds suspected of being colonized by *Candida* species requires assessment of the extent of extracellular matrix molecules that are present, because these extracellular matrix molecules can impair the antifungal efficacy of HOCl.

## Figures and Tables

**Table 1:** Log_10_ reductions in *Candida* species after 3 h exposure to 1% polygalacturonic acid + 0.8% caprylic acid and 400 ppm hypochlorous acid, compared to positive control, for 6 replicates of fibrin gel wound biofilm model.

	Tested Organisms				
	*C. tropicalis*	*C. glabrata*	*C. parapsilosis*	*C. albicans*	*C. auris*
Log_10_ of median viable colonies for control	9.75 × 10^6^	2.63 × 10^6^	1.71 × 10^7^	4.33 × 10^7^	1.72 × 10^4^
Log_10_ reduction of PG + CAP relative to control	3.85	5.02	4.88	5.76	4.16
Log_10_ reduction of HOCl relative to control	2.01	1.15	2.09	3.23	4.16
*p*-value of PG + CAP versus HOCl	0.014	0.014	0.015	0.015	>0.99

Statistical comparisons of CFU/mL values between PG + CAP and control, as well as between HOCL and control, were statistically significant for all *Candida* species tested (*p* ≤ 0.05).

## Data Availability

All data are available upon request.
